# The complete chloroplast genome of *Elytranthe Albida* (Loranthaceae), a hemiparasitic shrub

**DOI:** 10.1080/23802359.2019.1667911

**Published:** 2019-09-19

**Authors:** Xiaorong Guo, Zhijie Ruan

**Affiliations:** a Institute of Ecology and Geobotany, Yunnan University, Kunming, China;; b School of Ecology and Environmental Science, Yunnan University, Kunming, China

**Keywords:** Chloroplast genome, *Elytranthe albida*, Loranthaceae, hemiparasitic plant

## Abstract

The complete chloroplast genome (plastome) of *Elytranthe albida* (Blume) Blume (Loranthaceae) was sequenced. The plastome is 128,658 bp in length, which encodes 96 unique genes, including 64 protein-coding genes, 4 rRNAs, and 28 tRNAs. The complete plastome-based phylogeny indicated that *E. albida* is sister to remaining Loranthaceae species.

Although the chloroplast genomes (plastome) of the majority angiosperms are largely conserved in size, structure, gene content and arrangement, the lifestyle shift from autotrophy to heterotrophy leads to varying degrees of plastome degradation in parasitic plants. Compared with their autotrophic relatives, size reduction, physical or functional losses of genes, and structural rearrangement are often observed in parasitic plasomes (Wicke and Naumann 2018). Unfortunately, most parasitic plants (in particular hemiparasites) have not been sequenced the plastome (Wicke and Naumann 2018). The limited chloroplast genomic profiles in parasitic plants have hindered our understanding of the evolutionary pathway of plastome reduction associated with parasitism.

In this study, we sequenced the complete plastome of *Elytranthe albida* (Blume) Blume (Loranthaceae), a hemiparasitic shrub distributed in southwest China and Indochina (Qiu and Gilbert 2004). Specimens and silica gel dried leaf tissues were sampled from Tengchong, Yunnan, China (N24°59′06.86″, E98°34′37.87″). Voucher (JYH20180132) was deposited at the herbarium of Kunming Institute of Botany, Chinese Academy of Science (KUN). Genomic DNA was extracted from silica gel dried leaves. Purified DNA was sheared to fragments with an average length of 350 bp to construct Illumina shotgun library. The library was sequenced on Illumina HiSeq 2500 platform at BGI (Wuhan, China). The plastome of *Helixanthera parasitica* Loureiro (MG808038) was used as reference. The assembly Illumina reads into *E. albida* plastome was performed using the CLC genome assembler v. 4.0β (CLC Inc., Aarhus, Denmark). The Dual Organellar Genome Annotator database (Wyman et al. 2004) was used to annotate plastome. Transfer RNA (tRNA) genes were identified by tRNAscan-SE 1.21 (Schattner et al. 2005) with the default parameters. The validated plastome sequence of *E. albida* was deposited in the NCBI GenBank database under the accession number MN175256.

The *E. albida* plastome is 128,658 bp in length, which consists of a large single-copy region (72,966 bp), a small single-copy region (5250 bp), and a pair of inverted repeats (25,221 bp). It encodes 96 unique genes, including 64 protein-coding genes, 4 ribosomal RNAs, and 28 tRNAs. Compared with the plastome of *Erythropalum scandens* Blume (NC_036759), an autotrophic relative in Santalales, a total of 17 genes were deleted from the *E. albida* plastome, including 15 protein-coding genes (all the 11 *ndh* genes, *rps*15, *rps*16, *rpl*32, and *inf*A), and 2 tNNAs (*trn*G-UCC, and *trn*V-UAC).

The relationships of *E. albida* with other Loranthaceae species (Figure 1) were reconstructed by the phylogenetic analysis of complete plastome DNA sequences. The standard maximum likelihood (ML) method was used to reconstruct phylogenetic tree.

**Figure 1.**
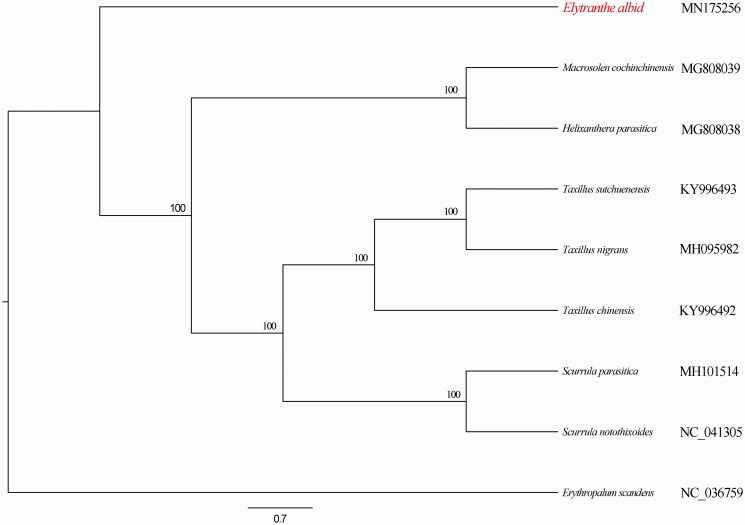
Phylogenetic relationships among Loranthaceae species, based on the ML analysis of complete plastomes. The number on each node indicates bootstrap percentage.

Sequences were aligned using MAFFT (Kazutaka and Standley 2013). ML analysis was conducted using RAxML-HPC BlackBox v8.1.24 (Stamatakis 2006) with 1000 replicates of rapid bootstrapping (BS) under the GTRGAMMAI model. The tree topology indicated that *E. albida* is sister to remaining Loranthaceae species (BS = 100%).
